# Inter- and Intraspecific Variation in *Drosophila* Genes with Sex-Biased Expression

**DOI:** 10.1155/2012/963976

**Published:** 2012-01-12

**Authors:** Lena Müller, Sonja Grath, Korbinian von Heckel, John Parsch

**Affiliations:** ^1^Department of Biology II, University of Munich (LMU), 82152 Planegg-Martinsried, Germany; ^2^Institute for Evolution and Biodiversity, University of Münster (WWU), 48149 Münster, Germany

## Abstract

Genes with sexually dimorphic expression (sex-biased genes) often evolve rapidly and are thought to make an important contribution to reproductive isolation between species. We examined the molecular evolution of sex-biased genes in *Drosophila melanogaster* and *D. ananassae*, which represent two independent lineages within the *melanogaster* group. We find that strong purifying selection limits protein sequence variation within species, but that a considerable fraction of divergence between species can be attributed to positive selection. In *D. melanogaster*, the proportion of adaptive substitutions between species is greatest for male-biased genes and is especially high for those on the X chromosome. In contrast, male-biased genes do not show unusually high variation within or between populations. A similar pattern is seen at the level of gene expression, where sex-biased genes show high expression divergence between species, but low divergence between populations. In *D. ananassae*, there is no increased rate of adaptation of male-biased genes, suggesting that the type or strength of selection acting on sex-biased genes differs between lineages.

## 1. Introduction

In sexually reproducing species, the evolution of reproductive isolation is closely coupled to the process of speciation. Indeed, the widely applied biological species concept defines species as “groups of actually or potentially interbreeding natural populations, which are reproductively isolated from other such groups” [1]. This definition has been of great utility to geneticists working with organisms like *Drosophila* that are separated into populations or species showing varying degrees of pre- and postzygotic reproductive isolation. The degree of isolation increases with the time since the species shared a common ancestor [2, 3]. 

Within species, prezygotic isolation is often observed as preferential mating of individuals (usually females) to other individuals from the same population. Such behavioral isolation has been observed for *Drosophila melanogaster* and *D. ananassae* populations that diverged within the past 15,000–20,000 thousand years [4, 5]. At the postzygotic level, it is often found that matings between closely related species produce hybrid offspring in which at least one sex (usually males) is either inviable or infertile. For example, species of the *D. simulans* complex, which diverged around 0.5–1.0 million years ago [6], produce viable hybrid offspring with only the males being infertile [7, 8]. Crosses between *D. melanogaster* and *D. simulans*, which diverged around 4-5 million years ago [6], produce viable, but sterile, offspring of only one sex (the sex of the *D*. *melanogaster* parent) [9, 10].

The observations from *Drosophila* suggest that the evolution of postzygotic reproductive isolation is a progressive process that involves the accumulation of incompatible alleles at many loci across the genome [11–13]. Since the first stage of isolation is typically hybrid male sterility, sequence divergence at genes involved in male reproduction is thought to be a major contributor to speciation [14]. Between the closely related species *D*. *simulans* and *D*. *mauritiana*, it is thought that *∼*60 loci contribute to hybrid male sterility [12]. To date, only a few of these loci have been mapped to the gene level [15–17]. For example, the first “speciation gene” identified between these two species, *OdsH*, encodes a homeodomain-containing transcription factor that is expressed in testis and shows extraordinary amino acid sequence divergence between *D*. *simulans* and *D*. *mauritiana *[15]. Within the homeodomain, 15 amino acids differ between these two *Drosophila* species, while only 17 amino acids differ between mouse and common ancestor of the *D*. *melanogaster* clade [15]. These findings suggested that the early stages of speciation are driven by the rapid adaptive evolution of genes involved in male reproduction [18]. Consistent with this, it has been found that genes known to be involved in male reproduction, but not directly implicated in reproductive isolation between species, evolve at a faster rate than other classes of genes in the genome [19–21].

With the advent of transcriptomic technologies, such as microarrays, it became possible to examine gene expression differences between males and females on a genomic scale. In *Drosophila*, a large fraction of genes differ in expression between the sexes [22]. Such genes are referred to as “sex-biased.” A meta-analysis over multiple experiments indicates that there are *∼*4,000 genes that show a large (greater than twofold) difference in expression between males and females of *D*. *melanogaster*, with *∼*2,000 showing male-biased expression and *∼*2,000 showing female-biased expression [23]. When statistical approaches are used to detect significant expression differences between the sexes, the number of sex-biased genes is even greater. For example, a meta-analysis with a false discovery rate of 5% classified 2,814 genes as male-biased and 4,056 genes as female-biased [23].

On average, male-biased genes display a faster rate of molecular evolution between species (as measured by the ratio of the nonsynonymous-to-synonymous substitution rates, *d*_N_/*d*_S_) than female-biased genes or genes with nearly equal expression in the two sexes (“unbiased genes”) [23, 24]. By comparing levels of polymorphism within species to divergence between species, it could be shown that male-biased genes undergo more adaptive evolution than female-biased or unbiased genes [25]. This pattern was especially pronounced on the X chromosome, where X-linked, male-biased genes show exceptionally high *d*_N_/*d*_S_ and the strongest signal of adaptive protein evolution [26]. Although species outside the *melanogaster* species subgroup have not been investigated as extensively, preliminary studies in *D. ananassae* and *D. pseudoobscura* suggest that male-biased expression does not have as much of an influence on evolutionary rate in these species as it does in *D*. *melanogaster* [27–29].

In this paper, we examine the molecular divergence of sex-biased genes within and between species using data from *D*. *melanogaster* and *D*. *ananassae*. We also investigate intra- and interspecific divergence at the level of gene expression. Our results indicate that much of the protein divergence observed between species is adaptive. Male-biased genes of *D*. *melanogaster*, especially those that reside on the X chromosome, show an exceptionally high rate of adaptation. However, these genes do not show unusually high sequence variation within or between populations. At the level of gene expression, we find that both male- and female-biased genes make a large contribution to expression differences between species but are underrepresented among genes that differ in expression between populations. These findings suggest that different selective forces contribute to interpopulation and interspecies divergence.

## 2. Materials and Methods

### 2.1. *D. melanogaster* Genes and Populations

In total, we analyzed DNA sequence polymorphism in 143 *D*. *melanogaster* genes (see Table  1 in Supplementary Material available online at doi:10.1155/2012/963976), which were classified as male-, female-, or unbiased in their expression using the Sebida database [23]. The numbers of sex-biased genes, as well as the numbers of X-linked and autosomal genes, are given in Table 1. All of the genes were sequenced in a sample of isofemale lines from two populations, one from Europe (Leiden, the Netherlands) and one from Africa (Lake Kariba, Zimbabwe) [34]. The number of alleles sequenced per population ranged from 7 to 12, with a mean of 11. Sequences of 136 of these genes were reported previously [24, 25, 35] and are available from the GenBank/EMBL databases under accession numbers AM293861–AM294919, AM998825–AM999334, and FM244915–FM246454. In addition, seven genes were newly sequenced for the current study and are available under accession numbers JN252131–JN252193 and JN374903–JN374992. For divergence calculations, a single allele from *D*. *simulans* was used [30].

### 2.2. *D. ananassae * Genes and Populations

**Figure 1:**
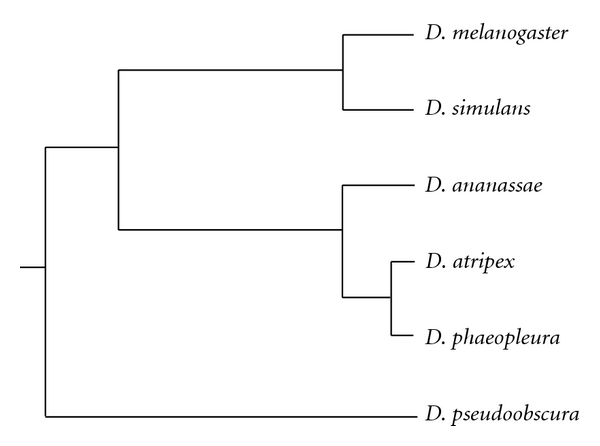
Phylogenetic relationship of the species used in this study [29, 30].

For *D*. *ananassae*, we surveyed polymorphism in 43 genes (Supplementary Table  1), which were classified as male-, female-, or unbiased in their expression using data from a custom amplicon microarray [29] and a whole genome microarray analysis [36]. The 43 genes were a subset of those analyzed in *D*. *melanogaster*. The numbers of sex-biased genes, as well as the numbers of X-linked and autosomal genes, are given in Table 1. All of the genes were sequenced in a sample of isofemale lines from Bangkok, Thailand [29], and the sequences are available from the GenBank/EMBL databases under accession numbers FN546265–FN546780. The number of alleles sequenced ranged from 8 to 12, with a mean of 11. To calculate divergence, a single allele from either* D. atripex* or *D. phaeopleura* was used. The phylogenetic relationship of the species is shown in Figure 1. 

### 2.3. DNA Sequencing

Genomic DNA was purified from single male flies, and target genes were PCR-amplified using protocols, primers, and cycling conditions described previously [25, 26]. Following PCR, the amplified products were purified with ExoSAP-IT (USB, Cleveland, OH, USA), and both strands were sequenced using BigDye version 1.1 chemistry and a 3730 automated sequencer (Applied Biosystems, Foster City, CA, USA). Sequences were edited using DNASTAR (Madison, WI, USA) and multiple alignments were generated using MUSCLE [37].

### 2.4. Statistical Methods

Standard polymorphism and divergence statistics were calculated using DnaSP version 5 [38]. To assess the significance of differences between sex-bias classes, the Kruskal-Wallis tests and the Mann-Whitney *U* tests were performed using R version 2.12.2 [39].

The distribution of fitness effects of new nonsynonymous mutations and the proportion of adaptive amino acid replacements between species, *α*, were estimated using the DoFE software [40]. For this, the shape parameter was set to 0.5 and the number of repetitions for the MCMC chain was set to 5,000,000. Prior to running, a look-up table was generated, setting the upper limit of *β* to 1 and the number of steps to 200. Otherwise, the default parameters were used. Synonymous sites were used as the neutral reference. This method requires the same sample size (number of sequences) for all genes. For *D*. *melanogaster*, we used a common sample size of 10 sequences from the African population. When more than 10 sequences were available for a gene, we randomly excluded surplus sequences. Genes with fewer than 10 sequences were excluded from the analysis. For *D. ananassae*, the above procedure was followed, but a common sample size of eight sequences was used.

## 3. Results

### 3.1. Data

In total, we analyzed DNA sequence polymorphism and divergence in 143 *D*. *melanogaster* and 43 *D*. *ananassae* protein-coding genes. Within each species, the genes could be assigned to one of three expression classes (male-, female-, or unbiased) on the basis of microarray data (Table 1) [23, 29, 36]. The proportion of genes in each expression class was similar, although there was a slight over-representation of male-biased genes. The genes could further be separated into those residing on the X chromosome and those residing on the autosomes (Table 1). For *D*. *melanogaster*, approximately one-third of the genes within each sex-bias class were X-linked. This allowed us to perform additional analyses in which X-linked and autosomal genes were considered separately within each expression class. Because the *D*. *ananassae* sample size was much smaller, we did not analyze X-linked and autosomal genes separately.

### 3.2. Selective Constraint on Sex-Biased Genes

**Figure 2:**
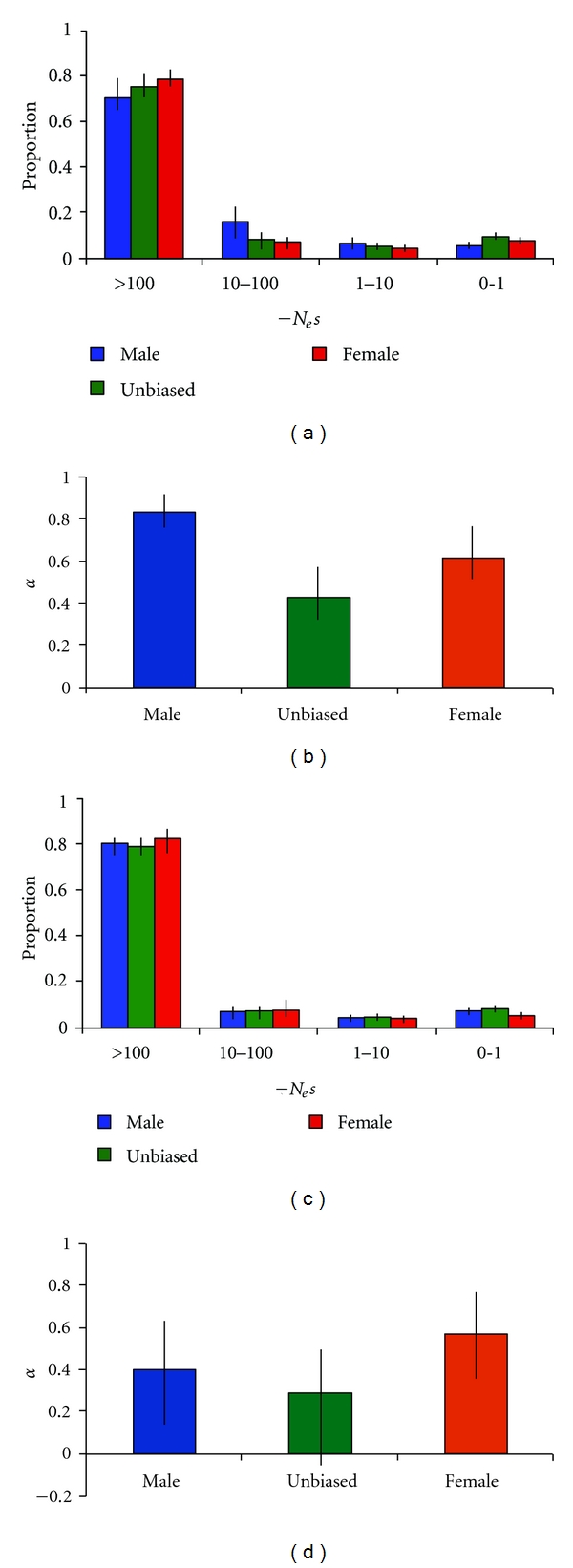
Distribution of fitness effects for nonsynonymous mutations within species and the proportion of adaptive nonsynonymous substitutions between species. Data for *D*. *melanogaster* are shown in (a) and (b), while those for *D*. *ananassae* are shown in (c) and (d).

To infer selective constraints, we used the method of Eyre-Walker and Keightley [40], which estimates the distribution of fitness effects of nonsynonymous mutations. In both *D*. *melanogaster* and *D*. *ananassae*, we found evidence for strong constraint on male-, female-, and unbiased genes, with the vast majority (>85%) of new mutations having a strongly deleterious effect, in which the product of the effective population size and the selection coefficient (*N*_e_s) is greater than 10 (Figure 2). Less than 10% of mutations fell within the neutral range (0 < *N*_e_s < 1). The level of constraint was similar across all classes of genes and in both species.

**Figure 3:**
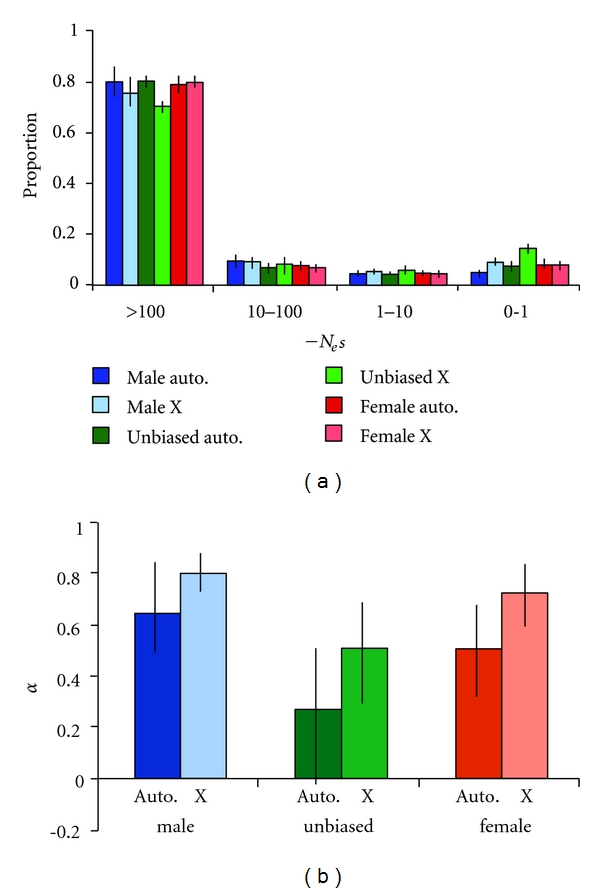
The distribution of fitness effects (a) and estimated proportion of adaptive substitutions (b) for autosomal and X-linked genes of *D*. *melanogaster*.

When the X-linked and autosomal genes of *D*. *melanogaster* genes were analyzed separately, there was again evidence for the predominance of strong purifying selection in all classes of genes (Figure 3). For male-biased and unbiased genes, there was a trend towards less constraint on the X chromosome. This pattern was not seen for female-biased genes.

### 3.3. Adaptive Evolution of Sex-Biased Genes

In both the* melanogaster* and *ananassae* lineages, we found that positive selection has made an important contribution to protein sequence divergence between species. For all classes of genes, the estimated proportion of adaptive nonsynonymous substitutions, *α*, ranged from 0.29 to 0.83 (Figure 2). The 95% confidence interval of *α* excluded zero in all cases, except for the unbiased genes of *D*. *ananassae* where it was −0.04 to 0.56. In *D*. *melanogaster*, male-biased genes had the highest mean *α* and its 95% confidence interval did not overlap with that of female-biased or unbiased genes, indicating a significantly greater proportion of adaptive substitutions in male-biased genes. This pattern was not seen for *D*. *ananassae*, where *α* was highest for female-biased genes (Figure 2), but the 95% confidence intervals of *α* overlapped among all classes of genes.

**Figure 4:**
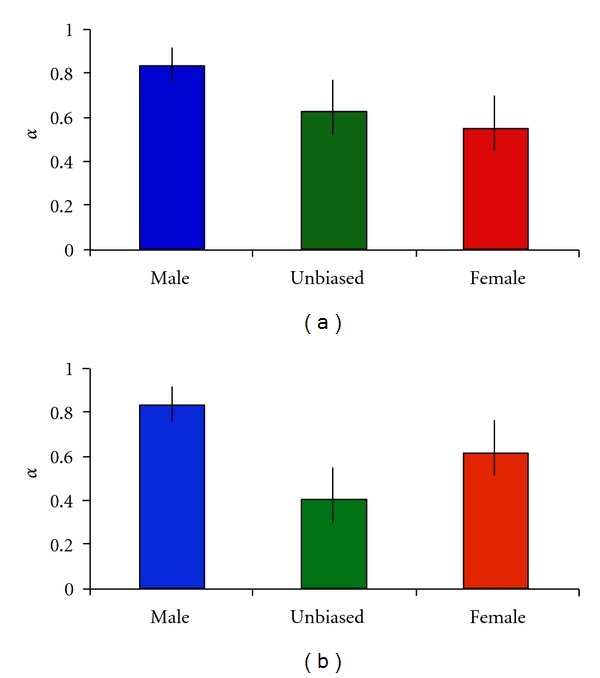
The estimated proportion of adaptive substitutions for *D*. *melanogaster* genes also present in the *D*. *ananassae* gene set (a) and for *D*. *melanogaster* genes that have an ortholog in *D*. *ananassae* (b).

Because the *D. ananassae* genes represented only a subset of those analyzed in *D. melanogaster*, it is possible that the observed differences in sex-biased gene evolution between species are a result of differences in gene composition or of limiting the *D*. *ananassae* genes to those that are well conserved and have identifiable orthologs in *D*. *melanogaster*. To examine these possibilities, we repeated our *D*. *melanogaster* analyses using only genes common to both species' gene sets (Figure 4(a)) or only genes with identifiable orthologs between species (Figure 4(b)). In both cases, we still observed higher values of *α* for male-biased genes than for female-biased or unbiased genes. For the set of common genes, which had a small sample size (37 genes total), the 95% confidence intervals of *α* overlapped among all classes of genes. However, for the set of genes with orthologs (108 genes total), the 95% confidence interval of *α* of male-biased genes did not overlap with that of female-biased or unbiased genes. This indicates that the increased level of adaptive evolution of male-biased genes in *D*. *melanogaster* is not attributable to the rapid evolution of young, newly evolved genes that lack orthologs in *D*. *ananassae*.

When *D*. *melanogaster* autosomal and X-linked genes were considered separately, there was a consistent pattern of higher *α* for X-linked genes of all classes, with the highest value observed for male-biased, X-linked genes (Figure 3). This pattern was even more pronounced when the nonsynonymous substitution rate was taken into account, as X-linked genes showed greater nonsynonymous divergence (Table 2).

### 3.4. Sequence Variation of Sex-Biased Genes within Populations

**Figure 5:**
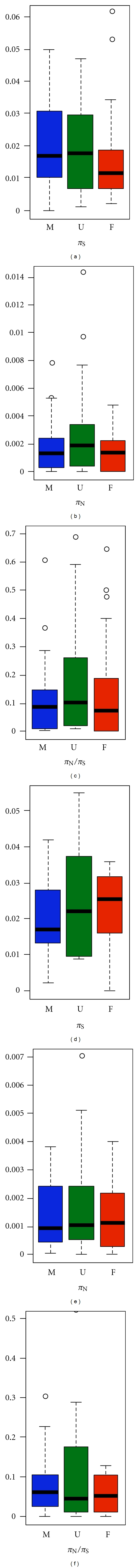
Intraspecies polymorphism in male-biased (M), unbiased (U), and female-biased (F) genes of *D*. *melanogaster* (a–c) and *D*. *ananassae* (d–f). Shown are distributions of synonymous nucleotide diversity (*π*_S_), nonsynonymous nucleotide diversity (*π*_N_), and their ratio (*π*_N_/*π*_S_). The *D*. *melanogaster* data are from the African (Zimbabwe) population. There were no significant differences among male-, female-, and unbiased genes in either species by any measure (the Kruskal-Wallis test, *P* > 0.10 in all cases).

Mean levels of nucleotide diversity (*π*) did not differ significantly among male-, female-, or unbiased genes in the Zimbabwe population of *D*. *melanogaster* or the Bangkok population of *D*. *ananassae* (Figure 5). This result held regardless of whether synonymous diversity (*π*_S_), nonsynonymous diversity (*π*_N_), or their ratio (*π*_N_/*π*_S_) was considered.

**Figure 6:**
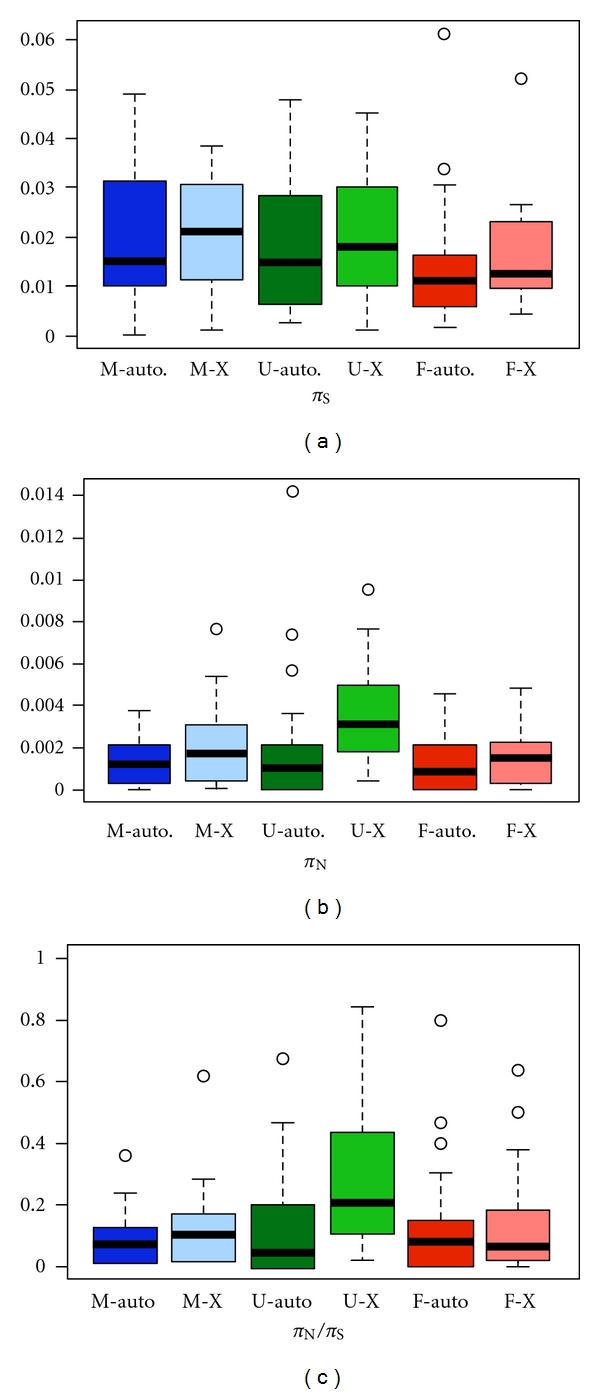
Intraspecies polymorphism in autosomal and X-linked genes of *D*. *melanogaster*. Shown are distributions of synonymous nucleotide diversity (*π*_S_), nonsynonymous nucleotide diversity (*π*_N_), and their ratio (*π*_N_/*π*_S_). The data are from the African (Zimbabwe) population. The only significant difference among expression classes was for *π*_N_ (the Kruskal-Wallis test, *P* = 0.03), where X-linked unbiased genes had significantly higher *π*_N_ than X-linked female-biased genes (the Mann-Whitney test, *P* = 0.01). Within expression classes, X-linked unbiased genes had significantly greater *π*_N_ and *π*_N_/*π*_S_ than autosomal unbiased genes (the Mann-Whitney test, *P* = 0.002 and *P* = 0.006, resp.).

When *D*. *melanogaster* X-linked genes were considered separately, there was a significant difference in *π*_N_ among male-, female-, and unbiased genes (the Kruskal-Wallis test, *P* = 0.03). This was mainly a result of X-linked, unbiased genes having high nonsynonymous diversity (Figure 6). There were no significant differences in *π*_S_, *π*_N_, or *π*_N_/*π*_S_ among autosomal male-, female-, or unbiased genes (the Kruskal-Wallis test, *P* > 0.20 in all cases). Within expression classes, there was consistently greater polymorphism at X-linked loci than at autosomal loci (Figure 6). This difference was significant only for unbiased genes, where *π*_N_, and *π*_N_/*π*_S_ were both greater on the X chromosome than the autosomes (the Mann-Whitney test, *P* = 0.002 and *P* = 0.006, resp.).

### 3.5. Sequence Divergence of Sex-Biased Genes between Populations

For *D*. *melanogaster*, we had sequence data for all 143 genes from both an African (Zimbabwe) and a European (the Netherlands) population, which allowed us to determine the contribution of sex-biased genes to interpopulation genetic differentiation. Two measures, *F*_ST_ and *D*_XY_ (the mean number of pairwise sequence differences between alleles of the two populations), indicated that there are similar levels of differentiation for male-, female-, and unbiased genes on both the X chromosome and the autosomes (Table 3). However, for all classes of genes, differentiation was greater at X-linked loci. For male- and female-biased genes, *F*_ST_ was significantly greater on the X chromosome when all sites or only synonymous sites were considered (Table 3). For unbiased genes, *D*_XY_ was significantly greater on the X chromosome for nonsynonymous sites (Table 3).

### 3.6. Intra- and Interspecific Divergence in Sex-Biased Gene Expression

**Figure 7:**
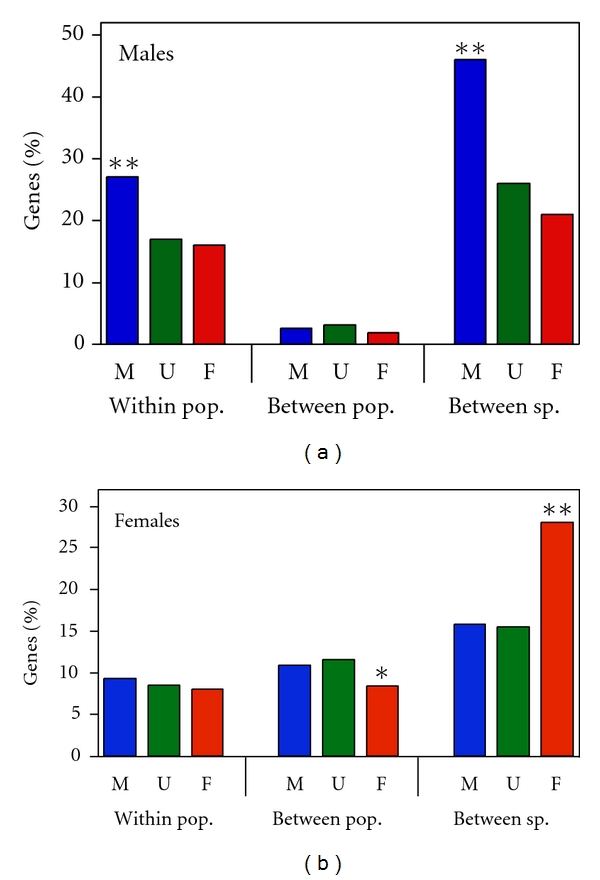
Gene expression variation within a population, between populations, and between species. Shown are the percentages of male-biased (M), unbiased (U), and female-biased (F) genes that show significant expression variation within a Zimbabwe population of *D*. *melanogaster*, between a Zimbabwe and a European population of *D*. *melanogaster* and between *D*. *melanogaster* and *D*. *simulans*. Expression variation was measured separately in males (a) and females (b). Data are from Hutter et al. [31], Müller et al. [32], and Ranz et al. [33]. Asterisks indicate significant differences from unbiased genes within the same comparison, as determined by Fisher's exact test. *P < 0.001, **P < 0.0001.

To determine the contribution of sex-biased genes to variation within and between species at the level of gene expression, we analyzed data from published microarray studies that investigated expression polymorphism within *D*. *melanogaster* [31, 32] and expression divergence between *D*. *melanogaster* and *D*. *simulans* [33]. Three types of expression variation were examined (intrapopulation, interpopulation, and interspecies) using data from males and females separately (Figure 7). When expression was measured in males, male-biased genes showed the highest levels of intrapopulation and interspecies divergence. However, male-biased genes did not show increased expression divergence between populations. When measured in females, female-biased genes showed the least intrapopulation and interpopulation expression polymorphism, but the greatest interspecies expression divergence.

## 4. Discussion

### 4.1. Selection on Sex-Biased Genes

Our analyses of polymorphism and divergence in *D*. *melanogaster* and *D*. *ananassae* uncovered several common patterns. First, there is strong purifying selection acting at the protein level in both species. We estimate that over 85% of all newly arising nonsynonymous mutations are deleterious. Second, a large proportion of amino acid substitutions that have become fixed between species can be attributed to positive selection. Our estimates of *α* range from 27 to 83% in *D*. *melanogaster* and 29–57% in *D*. *ananassae*. In *D*. *melanogaster*, male-biased genes showed the highest values of *α* (Figure 2), which is consistent with previous studies [25, 26]. In *D*. *ananassae*, there was no evidence for increased adaptive evolution of male-biased genes, which suggests that there are differences in sex-biased gene evolution among lineages [27, 29].

Our estimates of *α* are in line with previously published estimates and suggest that adaptive protein evolution is widespread across the *Drosophila* genus [41]. A recent study of *D*. *melanogaster* and *D*. *simulans* reported higher estimates of *α* for a randomly chosen (with respect to expression and function) set of genes [42]. However, this study was limited to X-linked genes, which tend to have higher values of *α* (Figure 3). This suggests that the use of only X chromosomal data may lead to an overestimate of the genome-wide proportion of adaptive substitutions.

### 4.2. Faster-X Evolution

Several factors could contribute to the increased rate of adaptive evolution of X-linked genes. For example, the X chromosome could have a larger effective population size (*N*_e_) than the autosomes. Assuming an equal sex ratio, the number of X chromosomes in a population is expected to be 75% that of the number of autosomes. However, sexual selection acting on males can lead to a reduction in the *N*_e_ of the autosomes relative to the X chromosome, and this could accelerate X chromosome evolution [43, 44]. In our populations of *D*. *melanogaster* and *D*. *ananassae*, which are thought to come from the ancestral species ranges [45, 46], the X chromosome and the autosomes have nearly identical *N*_e_ [29, 35, 47], making this explanation unlikely. Furthermore, there is no evidence for increased purifying selection on the X chromosome (Figure 3), which would be expected if it had a larger *N*_e_. This observation also argues against the possibility that an increased rate of recombination on the X chromosome leads to an increase in the efficacy of selection on X-linked loci by reducing interference among mutations [48, 49].

The accelerated rate of adaptive evolution on the X chromosome could also be explained by an increased rate of fixation of new, beneficial, recessive mutations due to their exposure to selection in hemizygous males [50, 51]. This would explain why the signal of adaptive evolution is strongest for male-biased genes, as they are expected to encounter selection mainly in the male (hemizygous) genetic background [26]. Although female-biased genes would be expected to receive the least benefit from selection in the male genetic background, a recent study found that mutations in female-biased genes often have phenotypic effects in males [52]. Thus, the increased rate of adaptive evolution seen for X-linked, female-biased genes could result from their exposure to selection in males.

### 4.3. Gene Expression Divergence

Our analyses of gene expression polymorphism and divergence revealed that the genes with the greatest expression divergence between species are those that are expressed predominantly in the sex that is used for comparison. When males are compared, male-biased genes show the greatest interspecific difference in expression (Figure 7). When females are compared, female-biased genes show the greatest interspecific difference in expression. These patterns are not seen for interpopulation expression divergence, where male- and female-biased genes consistently show less interpopulation expression divergence than unbiased genes, regardless of the sex that is compared (Figure 7). Thus, similar to protein divergence, gene expression divergence between species does not appear to be a simple extension of divergence between populations.

There are some caveats to our expression analysis. First, the experiments were performed by different groups, at different times, and with different microarray platforms. Thus, many factors may contribute to the differences seen among experiments. However, this problem will not apply to comparisons of male-, female-, and unbiased genes within each experiment, as all of the genes were measured together on the same microarrays. Thus, we expect comparisons of sex-biased genes within experiments to be robust. A second caveat is that the interspecies comparisons used only a single isofemale line of each species. This means that intraspecific polymorphism and interspecific divergence will be confounded. However, given the low level of expression polymorphism seen within species, it is unlikely that intra-specific gene expression polymorphism has much influence on measures of interspecific divergence. This is supported by the observation that, in females, there is no correspondence between the relative levels of expression polymorphism and divergence (Figure 7). However, for the experiments performed on males, we cannot exclude the possibility that the observed interspecific divergence of male-biased genes is inflated by intra-specific polymorphism.

### 4.4. Implications for Speciation

Although it is not possible to establish a direct link between sex-biased gene evolution and speciation in most cases, several of our observations are consistent with the rapid evolution of sex-biased genes (especially male-biased genes) contributing to reproductive isolation, at least for *D*. *melanogaster* and its close relatives. The male-biased genes examined here are expressed in reproductive tissues [26], and their rapid adaptive evolution could contribute to the male-hybrid sterility that is often seen as a first step in reproductive isolation. Furthermore, the rapid adaptive evolution of X-linked genes, especially those with male-biased expression, is consistent with the disproportionately large effect that the X chromosome has on hybrid breakdown [12, 13]. At the level of gene expression, male-biased genes make the largest contribution to the expression differences between species in males. Since the vast majority of male-biased genes are expressed in reproductive tissues [53], it is likely that expression differences also contribute to male hybrid sterility and other forms of hybrid breakdown.

## Supplementary Material

A list of all genes used in this study, along with their chromosomal locations and their sex-biased expression classifications, is provided in Supplementary Table 1.

## Figures and Tables

**Table 1:** Numbers of genes analyzed.

Species	Bias	Autosomal	X-linked
*D. melanogaster*	Male	35	18
	Unbiased	32	16
	Female	29	13

*D. ananassae*	Male	10	7
	Unbiased	9	5
	Female	10	2

**Table 2:** Rates of adaptive substitution in *D. melanogaster* and *D. ananassae*.

Species	Bias	Chrom.	*d*_N_^a^	*αd*_N_^b^
*D. melanogaster*	Male	Auto.	14.0	9.1
		X	60.8	49.8
	Unbiased	Auto.	8.3	2.3
		X	24.6	12.5
	Female	Auto.	16.1	8.3
		X	24.1	17.7

*D. ananassae*	Male	Auto. + X	17.7	7.1
	Unbiased	Auto. + X	15.9	4.6
	Female	Auto. + X	17.4	10.0

^a^Nonsynonymous substitutions per 1,000 nonsynonymous sites.

^b^Adaptive nonsynonymous substitutions per 1,000 nonsynonymous sites.

**Table 3:** Mean *F*_ST_ and *D*_XY_ between the African and European *D. melanogaster* populations.

Sites^a^	Bias^b^	Autosomal		X-linked	
		*F*_ST_ (SD)	*D*_XY_^c^ (SD)	*F*_ST_ (SD)^d^	*D*_XY_^c^ (SD)^d^
All	M	0.157 (0.142)	0.69 (0.35)	0.266 (0.141)**	0.72 (0.37)
	U	0.160 (0.125)	0.74 (0.60)	0.231 (0.231)	0.87 (0.48)
	F	0.195 (0.161)	0.56 (0.41)	0.343 (0.186)*	0.59 (0.30)

Syn	M	0.164 (0.164)	2.25 (1.87)	0.261 (0.151)*	2.68 (2.60)
	U	0.159 (0.139)	1.94 (1.63)	0.223 (0.020)	2.57 (1.56)
	F	0.185 (0.163)	1.54 (1.23)	0.319 (0.209)*	1.82 (1.27)

Non	M	0.090 (0.086)	0.14 (0.12)	0.185 (0.199)	0.27 (0.28)
	U	0.107 (0.123)	0.19 (0.27)	0.128 (0.143)	0.51 (0.80)**
	F	0.149 (0.171)	0.12 (0.15)	0.254 (0.305)	0.17 (0.17)

^a^“All,” all sites (including introns); “Syn,” synonymous sites; “Non,” nonsynonymous sites.

^b^“M,” male-biased; “U,” unbiased; “F,” female-biased.

^c^Mean pairwise differences between all African and all European sequences (in %).

^d^Asterisks indicate significant differences from autosomal genes by the Mann-Whitney test. *P < 0.05, **P < 0.01.
