# Supplementary material for: Inter- and Intraspecific Variation in Drosophila Genes with Sex-Biased Expression
Source: Int J Evol Biol. 2012 Jan 12;2012:963976. doi: 10.1155/2012/963976 (PMC3270394; doi:10.1155/2012/963976)
Supplement: Supplementary file 1 — A list of all genes used in this study, along with their chromosomal locations and their sex-biased expression classifications, is provided in Supplementary Table 1. [file 963976.f1.doc]

# Supplementary Table 1: Genes used for this study

|  | ***D. melanogaster*** | |  | ***D. ananassae*** | |
| --- | --- | --- | --- | --- | --- |
| **Gene** | **Bias** | **Chrom** |  | **Bias** | **Chrom.** |
| *CG1980* | Male | 3R |  |  |  |
| *CG2140* | Male | 2R |  |  |  |
| *CG3085* | Male | 2R |  | Male | 3L |
| *CG3483* | Male | 2R |  |  |  |
| *CG5045* | Male | 2L |  |  |  |
| *CG5276* | Male | 3R |  |  |  |
| *CG5565* | Male | 2L |  |  |  |
| *CG6036* | Male | 3R |  | Male | 2L |
| *CG6130* | Male | 3R |  |  |  |
| *CG6255* | Male | 3R |  |  |  |
| *CG6332* | Male | 3R |  |  |  |
| *CG6971* | Male | 3R |  | Male | 2L |
| *CG6980* | Male | 3R |  | Male | 2L |
| *CG7251* | Male | 2L |  |  |  |
| *CG7387* | Male | 3L |  | Female | 2R |
| *CG7409* | Male | 3L |  |  |  |
| *CG7929* | Male | 3R |  |  |  |
| *CG7931* | Male | 3R |  |  |  |
| *CG8277* | Male | 3L |  | Male | 2R |
| *CG8564* | Male | 3L |  |  |  |
| *CG9314* | Male | 2L |  |  |  |
| *CG9531* | Male | 2L |  |  |  |
| *CG10252* | Male | 3R |  | Male | 2L |
| *CG10307* | Male | 2R |  |  |  |
| *CG10750* | Male | 2L |  | Unbiased | 3R |
| *CG11037* | Male | 3L |  |  |  |
| *CG11475* | Male | 2R |  |  |  |
| *CG13527* | Male | 2R |  |  |  |
| *CG14717* | Male | 3R |  | Unbiased | 2L |
| *CG14926* | Male | 2L |  |  |  |
| *CG15179* | Male | 3R |  |  |  |
| *CG17376* | Male | 2L |  |  |  |
| *CG17956* | Male | 3R |  |  |  |
| *CG18266* | Male | 2L |  | Male | 3R |
| *CG18418* | Male | 3L |  | Male | 2R |
| *CG1314* | Male | X |  | Male | X |
| *CG1503* | Male | X |  |  |  |
| *CG1668* | Male | X |  |  |  |
| *CG1950* | Male | X |  |  |  |
| *CG2574* | Male | X |  |  |  |
| *CG2577* | Male | X |  | Male | X |
| *CG5334* | Male | X |  |  |  |
| *CG5662* | Male | X |  |  |  |
| *CG6789* | Male | X |  |  |  |
| *CG6999* | Male | X |  |  |  |
| *CG7860* | Male | X |  |  |  |
| *CG9156* | Male | X |  |  |  |
| *CG10920* | Male | X |  | Male | X |
| *CG11379* | Male | X |  | Male | X |
| *CG11697* | Male | X |  | Male | X |
| *CG12395* | Male | X |  |  |  |
| *CG12681* | Male | X |  |  |  |
| *CG18341* | Male | X |  | Unbiased | X |
| *CG1600* | Unbiased | 2R |  |  |  |
| *CG3476* | Unbiased | 2L |  | Male | 3R |
| *CG3652* | Unbiased | 2L |  |  |  |
| *CG3683* | Unbiased | 2R |  |  |  |
| *CG5915* | Unbiased | 3R |  | Male | 2L |
| *CG5919* | Unbiased | 3R |  |  |  |
| *CG6094* | Unbiased | 2L |  |  |  |
| *CG6913* | Unbiased | 3R |  |  |  |
| *CG6981* | Unbiased | 3L |  | Unbiased | 2R |
| *CG7484* | Unbiased | 3L |  |  |  |
| *CG7508* | Unbiased | 3R |  | Unbiased | 2L |
| *CG7953* | Unbiased | 2L |  |  |  |
| *CG8392* | Unbiased | 2R |  |  |  |
| *CG8844* | Unbiased | 2L |  |  |  |
| *CG9283* | Unbiased | 3L |  |  |  |
| *CG9437* | Unbiased | 2R |  |  |  |
| *CG9617* | Unbiased | 3R |  |  |  |
| *CG9822* | Unbiased | 2R |  |  |  |
| *CG9893* | Unbiased | 2R |  |  |  |
| *CG10035* | Unbiased | 3R |  | Female | 2L |
| *CG10623* | Unbiased | 2L |  |  |  |
| *CG10853* | Unbiased | 3L |  | Unbiased | 2R |
| *CG11785* | Unbiased | 3R |  |  |  |
| *CG11981* | Unbiased | 3R |  | Female | 2L |
| *CG13189* | Unbiased | 2R |  | Unbiased | 3L |
| *CG13419* | Unbiased | 3R |  |  |  |
| *CG13845* | Unbiased | 3R |  |  |  |
| *CG13934* | Unbiased | 3L |  |  |  |
| *CG16985* | Unbiased | 3L |  |  |  |
| *CG17404* | Unbiased | 3R |  |  |  |
| *CG31058* | Unbiased | 3R |  |  |  |
| *CG33976* | Unbiased | 3R |  |  |  |
| *CG1397* | Unbiased | X |  |  |  |
| *CG1751* | Unbiased | X |  |  |  |
| *CG2555* | Unbiased | X |  |  |  |
| *CG3603* | Unbiased | X |  |  |  |
| *CG9164* | Unbiased | X |  |  |  |
| *CG9571* | Unbiased | X |  |  |  |
| *CG9723* | Unbiased | X |  | Unbiased | X |
| *CG9919* | Unbiased | X |  |  |  |
| *CG11126* | Unbiased | X |  |  |  |
| *CG14227* | Unbiased | X |  |  |  |
| *CG14629* | Unbiased | X |  |  |  |
| *CG14772* | Unbiased | X |  |  |  |
| *CG14797* | Unbiased | X |  |  |  |
| *CG15247* | Unbiased | X |  |  |  |
| *CG15313* | Unbiased | X |  |  |  |
| *CG15336* | Unbiased | X |  | Unbiased | X |
| *CG1239* | Female | 3R |  | Unbiased | 2L |
| *CG2867* | Female | 3R |  |  |  |
| *CG3509* | Female | 3R |  | Female | 2L |
| *CG3831* | Female | 2R |  |  |  |
| *CG3975* | Female | 2L |  |  |  |
| *CG4236* | Female | 3R |  |  |  |
| *CG4299* | Female | 3R |  |  |  |
| *CG4570* | Female | 3R |  |  |  |
| *CG4973* | Female | 3R |  | Unbiased | 2L |
| *CG5272* | Female | 3L |  | Female | 2R |
| *CG5363* | Female | 2L |  |  |  |
| *CG5499* | Female | 3R |  | Female | 2L |
| *CG5757* | Female | 2R |  |  |  |
| *CG6459* | Female | 2R |  | Female | 3L |
| *CG6554* | Female | 3R |  |  |  |
| *CG6874* | Female | 3L |  |  |  |
| *CG7840* | Female | 2L |  | Female | 3R |
| *CG9135* | Female | 2L |  | Female | 3R |
| *CG9273* | Female | 2L |  |  |  |
| *CG9383* | Female | 3L |  | Unbiased | 2R |
| *CG10206* | Female | 2L |  |  |  |
| *CG12262* | Female | 3L |  |  |  |
| *CG12276* | Female | 3R |  | Female | 2L |
| *CG12314* | Female | 2L |  |  |  |
| *CG12909* | Female | 2R |  |  |  |
| *CG13690* | Female | 2L |  |  |  |
| *CG17361* | Female | 3L |  |  |  |
| *CG17950* | Female | 2R |  |  |  |
| *CG32409* | Female | 3L |  |  |  |
| *CG1749* | Female | X |  | Unbiased | X |
| *CG2222* | Female | X |  | Female | X |
| *CG3004* | Female | X |  | Unbiased | X |
| *CG3024* | Female | X |  | Male | X |
| *CG3704* | Female | X |  |  |  |
| *CG4593* | Female | X |  | Male | X |
| *CG8326* | Female | X |  |  |  |
| *CG8675* | Female | X |  |  |  |
| *CG9125* | Female | X |  |  |  |
| *CG9915* | Female | X |  |  |  |
| *CG12117* | Female | X |  |  |  |
| *CG14434* | Female | X |  |  |  |
| *CG15717* | Female | X |  | Female | X |
